# Bacterial meningitis in adults: a retrospective study among 148 patients in an 8-year period in a university hospital, Finland

**DOI:** 10.1186/s12879-023-07999-2

**Published:** 2023-01-23

**Authors:** Sakke Niemelä, Laura Lempinen, Eliisa Löyttyniemi, Jarmo Oksi, Jussi Jero

**Affiliations:** 1 Department of Otorhinolaryngology, Turku University Hospital and University of Turku, Savitehtaankatu 5, 20540 Turku, Finland; 2 Department of Radiology, HUS Medical Imaging Center, Helsinki University Central Hospital and University of Helsinki, Helsinki, Finland; 3 Unit of Biostatistics, Department of Clinical Medicine, University of Turku, Turku, Finland; 4 Department of Infectious Diseases, Turku University Hospital and University of Turku, Turku, Finland; 5 Department of Otorhinolaryngology, Head and Neck Surgery, Helsinki University Hospital and University of Helsinki, Helsinki, Finland

**Keywords:** Bacterial meningitis, Adults, Nosocomial, Glasgow Outcome Scale

## Abstract

**Background:**

Bacterial meningitis (BM) causes significant morbidity and mortality. We investigated predisposing factors, clinical characteristics, spectrum of etiological bacteria, and clinical outcome of community-acquired and nosocomial BM.

**Methods:**

In this retrospective study we analyzed data of 148 adults (age > 16 years) with BM treated in Turku University Hospital, Southwestern Finland, from 2011 to 2018. Besides culture- or polymerase chain reaction (PCR)-positive cases we also included culture-negative cases with laboratory parameters strongly suggestive of BM and those with meningitis-related findings in imaging. We used Glasgow Outcome Scale (GOS) score 1–4 to determine unfavorable outcome.

**Results:**

The median age of patients was 57 years and 48.6% were male. Cerebrospinal fluid (CSF) culture for bacteria showed positivity in 50 (33.8%) cases, although pre-diagnostic antibiotic use was frequent (85, 57.4%). The most common pathogens in CSF culture were *Streptococcus pneumoniae* (11, 7.4%), *Staphylococcus epidermidis* (7, 4.7%), *Staphylococcus aureus* (6, 4.1%) and *Neisseria meningitidis* (6, 4.1%). Thirty-nine patients (26.4%) presented with the triad of fever, headache, and neck stiffness. A neurosurgical procedure or an acute cerebral incident prior BM was recorded in 74 patients (50%). Most of the patients had nosocomial BM (82, 55.4%) and the rest (66, 44.6%) community-acquired BM. Ceftriaxone and vancomycin were the most used antibiotics. Causative pathogens had resistances against the following antibiotics: cefuroxime with a frequency of 6.8%, ampicillin (6.1%), and tetracycline (6.1%). The case fatality rate was 8.8% and the additional likelihood of unfavorable outcome 40.5%. Headache, decreased general condition, head computed tomography (CT) and magnetic resonance imaging (MRI), hypertension, altered mental status, confusion, operative treatment, neurological symptoms, pre-diagnostic antibiotic use and oral antibiotics on discharge were associated with unfavorable outcome.

**Conclusions:**

The number of cases with nosocomial BM was surprisingly high and should be further investigated. The usage of pre-diagnostic antibiotics was also quite high. Headache was associated with unfavorable outcome. The frequency of unfavorable outcome of BM was 40.5%, although mortality in our patients was lower than in most previous studies.

**Supplementary Information:**

The online version contains supplementary material available at 10.1186/s12879-023-07999-2.

## Background

Bacterial meningitis (BM) is a severe, life-threatening infection, which causes notable morbidity and mortality [1]. Meningitis has many etiologies: bacteria, fungi, viruses, and parasites or additionally it may be associated with cancerous conditions, medications or autoimmune diseases [2]. Worldwide number of BM cases may exceed 16 million cases, [3] with mortality up to 30% as estimated a few years ago [2, 4, 5].

BM can be classified into two groups; nosocomial (including postoperative BM) or community acquired [6]. The usage of conjugate vaccines during childhood against *Streptococcus pneumoniae*, *Neisseria meningitidis* and *Haemophilus influenzae B*, has significantly diminished the overall incidence of BM worldwide [7, 8]. *S. epidermidis* and *S. aureus* have been the main Gram stain positive cocci causing nosocomial meningitis [9]. Recently, however, the proportion of Gram stain negative bacteria such as *Acinetobacter baumannii, Klebsiella pneumoniae* and *Escherichia coli* has increased [10, 11]. BM cases rose between 2006 and 2016 with poverty being a strong predisposing factor [2]. The geographical location affects the incidence significantly though; in well-developed countries the incidence has been lately 0.5–1.5/100,000/year [12–14], but in developing countries the incidence may peak at even 1000/100,000 cases [2] due to epidemics [15–18].

The incidence is highest among young children and the elderly [6]. In the past 20 years the incidence, management and epidemiology of BM has changed [7]. The risk factors of BM consist of immunosuppression, human immunodeficiency virus (HIV)-infection [19], indoor air pollution [20], overcrowded houses [21], malnutrition [22] and sickle cell anemia [23]. The typical symptom triad of meningitis consists of headache, fever and meningismus [6]. Meningitis requires instant treatment and intense medical attention. In recent years the use of adjunctive dexamethasone with antibiotics has been associated to lower incidence of neurologic sequelae in survivors [6]. The diagnosis of meningitis is confirmed by cerebrospinal fluid (CSF) culture or polymerase chain reaction (PCR) on the CSF specimen [6]. Survivors of BM often present a variety of neurological difficulties afterwards, such as hearing loss and deafness, cognitive impairment, motor deficiencies, seizures, and paralysis [24].

Our aim was to study the epidemiology of BM in Southwestern Finland and the significance of possible predisposing factors and indicators of unfavorable outcome.

## Methods

The medical records of all patients over 16 years (n = 148) treated between 2011 and 2018 due to BM at Turku University Hospital, a tertiary referral center in the hospital district of Southwest Finland (480,000 inhabitants), were retrospectively reviewed.

We performed a database search with International Classification of Diseases 10th Revision (ICD-10)—codes for meningitis (Table 1 legend). From 747 hits we excluded all viral and aseptic meningitis by going through clinical findings of all patients, test results on blood and CSF specimens, and analysis of imaging data one by one. Finally, 148 adults were included with all types of BM: nosocomial and community-acquired. BM was defined nosocomial, if the patient was already admitted to hospital when developing BM or, if there was a history of surgery in the preceding 54 days. BM was defined community-acquired if the patient had no history of surgery or hospitalization during the preceding 54 days. Most of the published studies on BM has collected data on only CSF culture-positive meningitis, although it is known that a considerable proportion of BM cases may be culture-negative especially with antibiotics given pre-diagnosis [25]. In this study, besides culture- or PCR-positive cases we included cases with symptomatology of BM with neutrophilic pleocytosis and at least one of the following: decreased CSF glucose levels (< 2.2 mmol/L), high protein levels (> 1000 mg/L) or high CSF lactate levels (> 3.0 mmol/L), and those with meningitis-related findings in imaging. Neuroborreliosis cases were excluded. All methods of data collection remained consistent through 2011–2018.

**Table 1:** Baseline characteristics, underlying conditions, associated background infections, and signs and symptoms of patients with BM

Variable	BM patients n = 148
Demographics	
Age (years)	57.3 (median)
Men	72 (48.6%)
Finnish nationality	145 (98%)
Predisposing conditions	
Sinusitis	7 (4.7%)
Otitis	9 (6.1%)
Dental infection	9 (6.1%)
Neurosurgery-related infection	74 (50%)
Brain abscess	6 (4.1%)
Urinary tract infection	3 (2%)
Recurrent meningitis	7 (4.7%)
Smoking	21 (14.2%)
Alcohol overuse	18 (12.2%)
Sepsis	42 (28.4%)
Cancer	22 (14.9%)
Diabetes (type I and II)	17 (11.5%)
Hypertension	44 (29.7%)
Asthma	7 (4.7%)
Previous operative treatment	67 (45.3%)
Mental disability	1 (0.7%)
Respiratory infections	
Pneumonia	15 (10.1%)
Bronchitis	1 (0.7%)
Aspergillosis	1 (0.7%)
Upper respiratory	15 (10.1%)
Tonsillitis	1 (0.7%)
Skin infections	
Head area	11 (7.4%)
Other	11 (7.4%)
History of acute illness	
Seizures prior admission to hospital	8 (5.4%)
Pre-diagnostic antibiotic	85 (57.4%)
Pre-diagnostic corticosteroids	33 (22.3%)
Clinical findings at admission to hospital	
Decreased general condition	90 (60.8%)
Decreased consciousness	62 (41.9%)
Fever	123 (83.1%)
Headache	83 (56.1%)
Neck stiffness	73 (49.3%)
Triad of fever, headache, and neck stiffness	39 (26.4%)
Triad of fever, neck stiffness and altered mental status	20 (13.5%)
Vomiting	36 (24.3%)
Skin color change	12 (8.1%)
Confusion	58 (39.2%)
Aphasia or dysphasia	13 (8.8%)
Visual deviation	3 (2%)
Psychic retardation	3 (2%)
Pupil-asymmetry	3 (2%)
Babinski sign	2 (1.4%)
Nystagmus	2 (1.4%)
Eyes-fixed-gaze	2 (1.4%)
Hearing loss	1 (0.7%)
Dysarthria	1 (0.7%)
Vertigo	1 (0.7%)
Double vision	1 (0.7%)
Strabismus	1 (0.7%)
ICD-10 codes* for data search for BM	

*A87.9, B94.80, G00.9, G01*A32.1, G01*A39.0, G01*A69.2, G05.2*B83.2, A17.0, A32.1, A87, B01.0+, B02.1, B05.1+, B37.5, B38.4+, B45.1, B94.80, G00, G01, G02, G03, G05

A minimum of 2 ml CSF was gathered from adult patients with suspected BM. First, the appropriate chemical and cytological analyses were performed and a Gram staining was performed to screen the potentials bacterial pathogens. Then, the samples were centrifuged (2500*g*) for 15 min, and a drop of sediment was spread over culture media—chocolate agar plates and sheep blood agar plates. Moreover, a drop of sediment was added into fastidious anaerobe broth (FAB). Also, if postoperative BM was suspected, a fastidious anaerobe agar (FAA) plate was cultured. The aerobic media were incubated at 35 °C in 5% CO2 atmosphere. The aerobic plates and FAB were read on the first and second day after the inoculation. If no growth was detected, negative result was given. The FAA plates were read on the second and fourth day after the inoculation, after which the negative result was given if no growth was detected. The media were further incubated until total 7 days, and if growth was detected at that point, the clinician was informed.

When growth was detected on any media, the pathogens were identified with matrix-assisted laser-desorption-ionization time-of-flight (MALDI-TOF) mass spectrometry (MALDI Biotyper® System, Bruker Daltonics, Bremen, Germany).

Species-specific PCR-methods were not available for bacteria in our hospital. When requested by the clinician, general PCR for bacteria (16S rRNA—ribosomal ribonucleic acid-sequencing) was used to identify bacterial pathogens from CSF. Bacterial deoxyribonucleic acid (DNA) was isolated in our laboratory and sent for sequencing to Eurofins Genomics laboratory (Ebersberg, Germany) and the resulting DNA sequence was compared with BLAST-software (https://blast.ncbi.nlm.nih.gov) to GeneBank database.

Blood culture samples were collected into Bactec™ Plus Aerobic/F and Bactec™ Plus anaerobic/F bottles (BD Diagnostic Systems, Sparks, Maryland, USA). The bottles were incubated in Bactec™ 9240 or Bactec™ FX culture system (BD Diagnostic Systems), for 120 h or until signaled positive. The bacteria were identified by MALDI Biotyper® System (Bruker Daltonics, Bremen, Germany).

Disk diffusion, minimum inhibitory concentration (MIC) gradient -method and the VITEK® 2 Compact automated ID/AST system (bioMerieux E-test) were used to analyze susceptibility to variety of antibiotics. The results were interpreted in accordance with the European Committee on Antimicrobial Susceptibility Testing (EUCAST) guidelines.

Imaging data was interpreted by radiologists. Patients with meningitis-related findings in imaging according to radiologists were included.

We defined a scale on outcome with Glasgow Outcome Scale (GOS-1 = death, 2 = vegetative state—unable to interact with the environment, 3 = severe disability—unable to live independently, 4 = moderate disability—can live independently but unable to return to previous work, 5 = mild or no disability) at discharge. Unfavorable outcome was chosen to be scores 1–4.

### Statistical analysis

The categorical variables are summarized with counts and percentages (%), continuous variables with range, mean for normally distributed variable or median otherwise.

To find out factors affecting to unfavorable outcome (GOS scores 1–4), log binomial model was performed separately for each factor (univariate approach) and reported with relative risk (and it’s 95% confidence intervals, CI) together with p-value. All tested factors are presented in Additional file 1.

Association with two categorical variables was tested with Fisher’s exact test.

The data analysis was generated using SAS software, Version 9.4 of the SAS System for Windows (SAS Institute Inc., Cary, NC, USA).

## Results

### Background

**Fig. 1:**
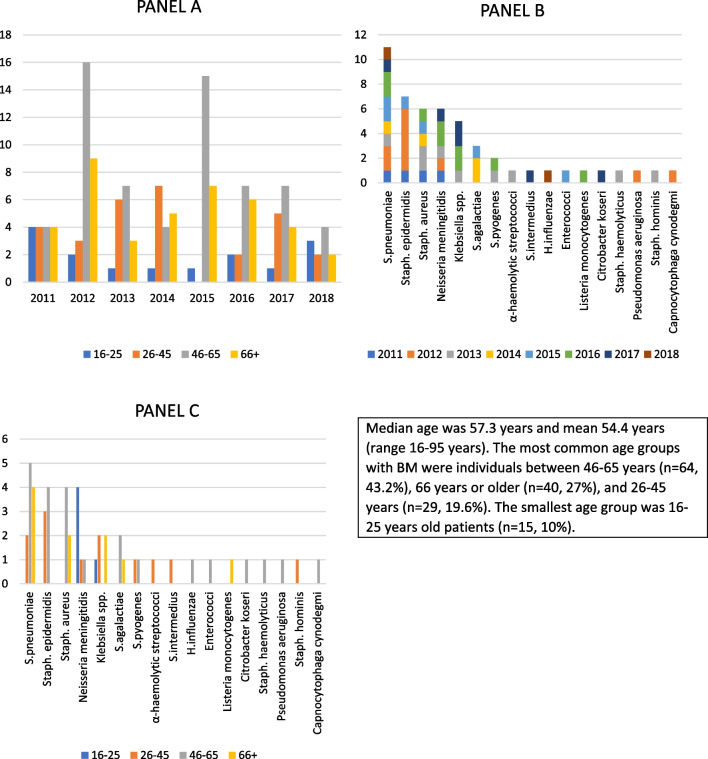
**A** Incidence of bacterial meningitis. **B** Bacteria cultured from CSF by year of meningitis. Panel **C**. Bacteria from CSF culture by age groups

The medical records of 148 adults with BM were included in this study. There were 72 (48.6%) males and 76 (51.4%) females. Median age was 57.3 years (range 16–95). Almost all (145, 98%) of patients were of Finnish nationality. Baseline characteristics, underlying conditions, associated background infections, and signs and symptoms of patients on admission are presented in Table 1. The age distribution is presented in Fig. 1.

Most patients were first contacted with hospital emergency department (120, 81.1%) and some to local health center (24, 16.2%) and a few with private medical clinics (4, 2.7%). Most individuals had nosocomial BM (82, 55.4%) and the rest (66, 44.6%) community-acquired BM. Neurosurgical procedure or acute cerebral incident prior BM were seen in 74 patients (50%). Forty-nine patients (33.1%) had no previous infection or operation. Nine patients (6.1%) had otogenic, another 9 patients (6.1%) odontogenic, and seven patients (4.7%) sinonasal etiology of BM. Seven patients (4.7%) had recurrent BM. Almost all cases were diagnosed in the hospital district of Southwest Finland (139, 93.9%), but 9 patients (6.1%) were transferred to our hospital from regional hospitals elsewhere. Vaccination coverage was poorly mentioned in the patient records, only one (0.7%) was reported to having received all vaccinations in the Finnish national vaccination program.

Active cancer, diabetes, lower and upper respiratory infections, excessive alcohol use, smoking, and skin infections were frequent (Table 1.) Sixty-seven patients (45.3%) had previous surgery and two (1.4%) of them were ear related.

### Clinical picture

The median length of symptoms before admission to hospital was one day (mean 2.34; range 0–30 days). Eight patients (5.4%) had previous seizures (3 generalized, 1 focal, 4 undefined) prior admission to hospital. In the emergency department 8 (5.4%) patients had seizures. Decreased level of consciousness was observed in 62 (41.9%) patients, 4 of them being in coma—one in a ventilator and three sedated due to difficult general condition. Eighteen patients (12.2%) had two or three neurological symptoms simultaneously. One (0.7%) monoparesis and two (1.4%) hemiparesis were observed, but those were related to acute ischemic brain attack prior to BM. Skin color changes were observed in 12 (8.1%) patients: petechiae in 9, marble skin in 2 patients (1.4%), and in one patient yellowish skin. All signs and symptoms are presented in Table 1.

### Laboratory results

Blood, plasma, and CSF laboratory results are presented in Table 2.

**Table 2:** Values of fundamental laboratory results at admission to hospital

Laboratory results	Median	Normal values
C-reactive protein (mg/L)	61	< 10
Leukocyte count in blood (× 10^9^/L)	12.2	3.4–8.2
Plasma glucose (mmol/L)	6.0	< 6.0
Leukocyte count in CSF (× 10^6^/L)	566	0–5
Granulocyte percentage in CSF (%)	75	0
Glucose levels in CSF (mmol/L)	1.5	2.2–4.2
Lactate levels in CSF (mmol/L)	5.7	1.1–2.2
Protein levels in CSF (mg/L)	1563	150–650

### Causative pathogens

Blood culture for bacteria was positive with 42 (28.4%) patients. CSF culture for bacteria was performed in 146 (98.7%) cases showing positivity in 50 (33.8%) cases. PCR of CSF samples was performed with 46 individuals (31.1%) showing positivity in 10 (20.4%) cases. Same pathogen in CSF and in blood was detected in 17 (11.5%) individuals. The most common pathogens were *S. pneumoniae* (11, 7.4%), *S. epidermidis* (7, 4.7%), *S. aureus* (6, 4.1%), *N. meningitidis* (6, 4.1%) and *Klebsiella* spp. (5, 3.4%). *S. epidermidis* was defined as a causative pathogen of BM if the patient had BM symptoms, CSF culture or PCR was positive for *S. epidermidis* and there was simultaneous CSF pleocytosis. Culture method was used to identify seven *S. epidermidis* cases and PCR was used once.

There were nine (6.1%) Gram stain negative rods cultured from CSF in our study: Five *Klebsiella *spp, one each *H. influenzae, Citrobacter koseri, Pseudomonas aeruginosa* and *Capnocytophaga cynodegmi. Pasteurella multocida* was detected once with PCR.

All causative pathogens cultured from CSF are presented in Fig. 1. Besides from CSF, bacteria were cultured from other sources. Most common pathogens retrieved from blood were *S. pneumoniae* (12, 28.6%), *S. aureus* (7, 16.7%), *S. pyogenes* (5, 11.9%), *N. meningitidis* (4, 9.5%), *S. epidermidis* and *S. agalactiae* (both 3, 7.1%). *Klebsiella *spp, *H. influenzae*, *S. salivarius, Anaerobic streptococci, E. cloacae, P. multocida, Staphylococcus pettenkoferi*, and *A. baumannii* were all detected once.

Pathogens most cultured from pus (from ear, sinuses, neurosurgical wound, or abscess) were *S. aureus* (3, 17.6%) and *S. pyogenes* (2, 11.8%). *S. pneumoniae, S. epidermidis, Klebsiella *spp*, S. agalactiae, α-haemolytic streptococci, S. intermedius, H. influenzae, P. aeruginosa, E. cloacae, Raoultella *spp,* Eikenella corrodens* and *Serratia marcescens* were all detected once.

*Staphylococcus epidermidis* (4, 66.7%) was responsible for most cultured pathogens from intracranial material. *Propionibacterium* and *S. aureus* were detected once.

Intracranial material was defined as cannula, shunt, or suture. In 7 (4.7%) cases the bacterium was detected by PCR from a CSF specimen when culture was negative; *N. meningitidis* (3), *S. epidermidis* (1), *P. multocida* (1), *Cellulosimicrobium* (1) and *Bacillus cereus* (1). In three (2%) cases two different pathogens were simultaneously detected in the CSF.

### Antibiotic resistance

**Fig. 2:**
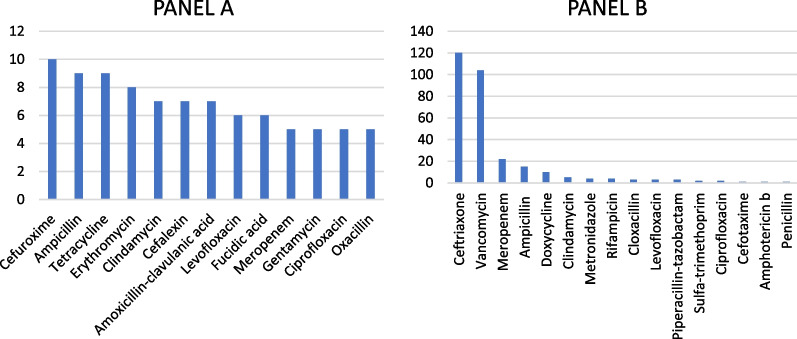
**A** Antibiotic resistant bacterium species cultured from CSF. **B** Quantity of patients with antibiotics used intravenously after the diagnosis of bacterial meningitis

CSF pathogens causing BM were most likely resistant to cefuroxime (10, 6.8%), ampicillin (9, 6.1%) and tetracycline (9, 6.1%). All CSF culture resistance profiles are shown in Fig. 2 Panel A. Seven (4.7%) bacteria from CSF were resistant to one antibiotic, three (2%) to two different antibiotics, one (0.7%) to three different antibiotics, five (3.4%) to four different antibiotics, two (1.4%) to six different antibiotics, three (2%) to eight different antibiotics, three (2%) to 11 different antibiotics and one (0.7%) to 12 different antibiotics. It is worth noticing that there were no species of bacteria resistant to vancomycin or ceftriaxone. Multi-drug resistance (resistance to three or more antibiotics) was seen in 13 (8.8%) patients, mostly patients with nosocomial BM (8, 5.4%).

In other samples (blood, pus or intracranial material), 30 (20.3%) cases presented resistance to at least one antibiotic was detected. More specifically, 13 (8.8%) bacterial species were resistant to one antibiotic, and 4 (2.7%) to two, 2 (1.4%) to three, 5 (3.4%) to four, 2 (1.4%) to six, and 2 (1.4%) to seven different antibiotics. On one occasion each, resistance was detected to eight, nine and 11 different antibiotics. There were six (4%) coexistent fungal infections.

### Imaging

On admission, head CT was performed to most patients (119, 80.4%) and MRI to 26 (17.6%) patients. In 14 (9.5%) cases imaging findings were consistent with meningitis, e.g., leptomeningeal or pachymeningeal intensification, most frequently seen with MRI (8, 57.1%), but also with CT (6, 42.9%), presenting specificity of 30.8% with MRI and 5.0% with CT. In 15 (10.1%) patients imaging showed findings consistent with elevated intracranial pressure. Imaging controls were performed most frequently by MRI (62, 41.9%). CT controls were performed to 43 (29.1%) patients. Fifty (33.8%) patients had at least once imaging control performed 1 month to two years after discharge from hospital.

### Treatment

Pre-diagnostic antibiotics, including perioperative ones, were given to 85 (57.4%) patients. Of them, 49 (57.6%) suffered from postoperative meningitis.

Pre-diagnostic corticosteroids were used with 33 (22.3%) patients: tablets, intravenous products, inhalators, nasal sprays, and intravenous products, in 19, 10, 3, and 1 patient, respectively. The most frequent corticosteroid used was dexamethasone (12, 8.1%) followed by hydrocortisone, betamethasone, prednisolone, fluticasone propionate, methyl prednisolone, mometasone furoate and fludrocortisone, with 5, 5, 4, 3, 2, 1, and 1 patient, respectively. In 23 (15.5%) patients’ treatment with acyclovir was initiated on admission.

After the diagnosis of BM, ceftriaxone was the most frequently used empiric antibiotic regimen (117 cases, 79.1%), followed by meropenem and vancomycin with 11 (7.4%) cases each. Altogether 11 different antibiotics were used as a first choice. The most used second antibiotic in conjunction with the first one was vancomycin (93, 62.8%), meropenem (11, 7.4%), doxycycline (8, 5.4%), ampicillin and clindamycin (both 5, 3.4%). Antibiotic monotherapy was given to 13 (8.8%) patients. Eighteen patients received three-modal antibiotic therapy with the most common third antibiotic being ampicillin (8, 5.4%). The total number of intravenous antibiotics used are shown in Fig. 2. Ceftriaxone and vancomycin were the most used empiric antibiotics among all occasions and so were them also after confirmed etiology.

The most common number of different concomitant antibiotics used were three (50, 33.8%), two (34, 23%), four (22, 14.9%), five (19, 12.8%), seven (8, 5.4%), six (6, 4.1%), eight and nine (both 3, 2%), one (2, 1.4%) and ten (1, 0.7%). The numbers include perioperative, intravenous, and in some cases oral antibiotics at discharge.

After the diagnosis of BM, corticosteroids were used in 79 patients (53.4%). Operative treatment was required for 56 (37.8%) patients with most cases being neurosurgical. Mastoidectomy was performed to six (4.1%) patients of whom all had otogenic meningitis.

The median duration of intravenous antibiotic treatment was 18 days (range 2–125). At discharge, 29 (19.6%) patients were prescribed oral antibiotics with the duration ranging commonly from 5 to 30 days, and in a few patients for 100–270 days as a suppressive antibiotic treatment for various reasons. Five most common oral antibiotics used were amoxicillin clavulanic acid (5, 3.4%) followed by clindamycin, moxifloxacin, penicillin and cefalexin (all 4, 2.7%). Median value of the days on antibiotics (combined all intravenous and oral) was 21 days.

### Outcome

The median length of hospital stay was 20 days. Neurological sequelae after BM a total of 49 patients (33.1%) had developed at least one neurological deficit at the time of discharge. Most common deficits were memory difficulties (15, 10.1%), mental regression (13, 8.8%), dysphasia or aphasia (9, 6.1%), visual disorders (9, 6.1%), vertigo and hydrocephalus (7, 4.7% each), decreased level of consciousness (5, 3.4%), hemiparesis and change in personality (4, 2.7%). Twelve (8.1%) patients had their hearing checked with audiogram during their hospital stay. In the period from the moment of discharge to one-year control visit, eight (5.4%) patients had permanent hearing impairment. There was no deafness diagnosed in any patient. Naturally, most of the neurological sequelae were related to neurosurgical procedures.

Fourteen patients (9.5%) died (GOS score 1), all but one directly to BM. From the survivors 5 (3.4% of all patients), 18 (12.2%), 23 (15.6%) and 89 (60.1%) patients had the GOS score 2, 3, 4, and 5, respectively. In total, 40.5% (60/148) had unfavorable outcome (GOS scores 1–4). 30-day all-cause mortality was 10.8% with one-year and two-year overall mortality being 14.2% and 19.6%, respectively.

Patients who suffered from otogenic meningitis had unfavorable outcome likelihood of 22.2%, those from sinonasal meningitis 28.6%, from odontogenic meningitis 33.3%, from neurosurgery-related meningitis 44.6% and patients with no specific source of infection 38.8%. CSF-culture appeared to be most frequently negative with neurosurgery-related meningitis with 16/58 (21.6%) individuals.

Headache (p = 0.0001, 95% CI 0.16–0.35), decreased general condition (p = 0.0001, 95% CI 0.23–0.67), head CT (p = 0.0001, 95% CI 0.073–0.64) and MRI (p = 0.040, 95% CI 0.92–4.0), hypertension (p = 0.0002, 95% CI 0.34–0.70), altered mental status (p = 0.0002, 95% CI 0.47–0.73), confusion (p = 0.0011, 95% CI 0.36–0.78), operative treatment (p = 0.012, 95% CI 0.42–0.89), neurological symptoms (p = 0.023, 95% CI 0.44–0.93), pre-diagnostic antibiotic use (p = 0.026, 95% Cl 0.40–0.97) and oral antibiotics on discharge (p = 0.039, 95% CI 0.94–3.6) were correlated with unfavorable outcome.

All statistical analyses are shown in Additional file 1.

## Discussion

This study shows extensively the variety of clinical picture, pathogens, and outcome of infection with different etiologies of BM in Southwestern Finland. Our study included all types of meningitis, culture-positive and -negative cases of BM, covering larger entities than if only culture-positive meningitis were included [25]. Due to severity of the disease it’s essential to regularly evaluate possible predictors of unfavorable outcome.

*S. pneumoniae* was the most frequently (11, 7.4%) detected pathogen confirming the results of earlier studies [26, 27]. In addition to *S. pneumoniae*, *N. meningitidis* is a common causative pathogen of BM in all age groups presented earlier [28], but in our study the *N. meningitidis* cases were most commonly seen with young adults aged 16–25 years. Surprisingly there were only one *Listeria monocytogenes* and *H. influenzae* meningitis although it is shown that these pathogens cause around 9% and 7% of BM worldwide, respectively [6]. Still, it is possible that patients with septicemia and concomitant meningitis had an ICD-10 diagnosis number of only sepsis in their patient records.

We presented median CSF leucocyte count of 566 × 10^6^/L and median protein levels of 1563 mg/L. However, even normal CSF leucocyte levels can indicate BM, especially combined with high protein levels. In those cases, the outcome may be even worse than normally, with incidence of unfavorable outcome even 59% and mortality 31% [29].

In 2011 to 2018 we found seven patients with culture-negative CSF specimens positive for bacteria with PCR, a technique shown to be a lot more sensitive than culture [25]. CSF culture has shown varying sensitivities of 43–85%, and a specificity up to 97%, at least in patients without the use of pre-diagnostic antibiotics [30–32]. The use of antibiotics reduces the identification of pathogens at least by 30% [33]. Compared to culture, multiplex or quantitative PCR has shown up to two-fold better sensitivities and specificities up to 100% [31, 33]. Our results of CSF culture positivity (33.8%) are compatible with previous studies, especially with frequently used pre-diagnostic antibiotics (57.4%). Our 16sRNA based PCR method presented clearly inferior to newer methods described below. CT was used with most patients (80.4%) and MRI with only 17.6% of patients, which may be due to lack of resources for use of MRI-equipment and the need for quicker results. Our results presented 30.8% and 5.0% specificity with MRI and CT, respectively, on identifying meningitis-related findings in imaging. Previous study indicated MRI being more specific but with a lower specificity of 16% [34].

Previous studies have shown that CSF sterilization may occur in hours after using parenteral antibiotics. Meningococci may be sterilized within 2 h and pneumococci within 4 h after administering parenteral antibiotic therapy [35]. Our results showed pre-diagnostic antibiotic use correlated with both negative blood- and CSF culture. Positive blood culture was correlated with positive CSF culture. Therefore, in some cases pre-diagnostic administration of antibiotics before lumbar puncture may cause lack of detectable bacteria despite BM. However, in emergency situations such as sepsis and suspicion of BM the fast administration of antibiotics is essential [36].

Triad of fever, neck stiffness and altered mental status has been previously reported with 41–59% BM cases [37, 38]. In our study, the prevalence of the presentation with this triad was much lower (13.5%). However, another triad—fever, neck stiffness and headache—was more frequent (39, 26.4%). Therefore, it is clear that the absence of the classical triad cannot be used to rule out the possibility of BM. In an older study headache was not reported to be correlated with unfavorable outcome [26], but with a more recent study [37] headache was correlated with unfavorable outcome, as was the case also in our study. Therefore, clinicians should pay even more attention to BM patients suffering from headache and not only altered mental status, which may be a sign of a more advanced disease.

The treatment administered after the diagnosis of BM remained highly efficacious due to the lack of resistance of bacteria to the most used antibiotics ceftriaxone and vancomycin, as proved earlier with ceftriaxone [37].

Community-acquired BM has been shown to cover most of BM cases with even proportion of 86%, with pneumococci being responsible for most of the episodes in adults. Nosocomial BM, on the other hand, has been shown to cover varying proportions of all BM with 14%-73%, most cases being staphylococci dominant. Nosocomial BM cases has seen an increase during the conjugate-vaccine era [39–42]. Insufficiency of antibiotic prophylaxis in neurosurgical operations may explain present considerable proportion of individuals with nosocomial BM [41].

The number of cases with nosocomial BM was relatively high. This is planned to be a topic of our further research. Pre-diagnostic antibiotic use seems to be linked with unfavorable outcome. This may be due to preoperative antibiotics given to patients with neurosurgery and the operative risks. Neurological symptoms and confusion were associated with unfavorable outcome, as presented earlier [39]. Severe symptoms on admission require more often imaging to exclude other disorders, therefore relating both logically to unfavorable outcome. Oral antibiotics prescribed at discharge were also correlated with unfavorable outcome, probably due to more severe clinical picture and the need for longer antibiotic therapy.

The frequency of unfavorable outcome of BM being 40.5% in our study was compatible with previous research showing the frequency of 38% [26], but this previous European study excluded all cases with nosocomial meningitis. Previous studies have reported overall mortality of 10–17% [26, 27], but in our study the mortality was only 8.8%. A recent study [37] from Lithuania presented likelihood of unfavorable outcome (GOS 1–3 in their study) to be 15.7% and a mortality (GOS 1) of 5.7%. For comparison, the respective proportions in our study were 24.3% for GOS 1–3 and mortality (GOS 1) of 8.8%. However, straight comparison cannot be done, since we also included patients with nosocomial BM.

Nosocomial meningitis requires often surgical intervention with significant risks. Therefore, it could be interpreted that our results of unfavorable outcome are compatible with previous studies [37, 39, 43].

The worldwide disease burden of BM is high especially in developing countries. Prevention of the disease with vaccines falls behind many other vaccine-preventable notorious diseases. Despite good progress of vaccine development against pathogens of BM, corresponding figures of measles (93.0%) and tetanus (90.7%) vaccination coverage imply that against BM also this could be better [44].

Our study has limitations. The fact that it was a single center study, is a limitation due to somewhat small number of patients, but the design allows uniform data collection and reliable transfer to analyses. Retrospective nature of this study may have caused inaccurate data collection in some cases. Our patients may not represent the whole population of patients in Finland. Also, we are unable to exclude the possibility of neurosurgery itself causing unfavorable outcome on patients. In the future, comprehensive prospective studies are needed to better determine prognostic factors of BM.

## Conclusions

*Streptococcus pneumoniae* was the most frequent causative pathogen of BM in our study. The proportion of nosocomial BM was surprisingly high, and so was the use of pre-diagnostic antibiotics. Ceftriaxone and vancomycin were the most used antibiotics, and no pathogen presented resistance to them. Headache was associated with unfavorable outcome. Pre-diagnostic antibiotic use predicted unfavorable outcomes, but the reasons may be multifactorial.

The likelihood of unfavorable outcome was compatible with previous studies. However, mortality in our patients was lower than in most previous studies.

The need for developing vaccines against wider spectrum of pathogens causing BM remains of utmost importance. Further research is needed on risk factors, pre- and perioperative antibiotic prophylaxis, and knowledge on different causative pathogens of meningitis to specify appropriate treatments, to recognize special populations, and to improve recovery of patients with BM [2].

## Supplementary Information

**Additional file 1: Table S1.** All statistical analyses performed.

## Data Availability

The datasets generated during the current study are available from the corresponding author on reasonable request.

## Abbreviations

BM: Bacterial meningitis
HIV: Human immunodeficiency virus
CSF: Cerebrospinal fluid
PCR: Polymerase chain reaction
ICD-10: International Classification of Diseases 10th Revision
CRP: C-reactive protein
GOS: Glasgow Outcome Scale
MALDI-TOF: Matrix-assisted laser-desorption-ionization time-of-flight
FAA: Fastidious anaerobe agar
FAB: Fastidious anaerobe broth
MIC: Minimum inhibitory concentration
EUCAST: European Committee on Antimicrobial Susceptibility Testing
rRNA: Ribosomal ribonucleic acid
DNA: Deoxyribonucleic acid
CT: Computed tomography
MRI: Magnetic resonance imaging
