# Supplementary material for: Bacterial meningitis in adults: a retrospective study among 148 patients in an 8-year period in a university hospital, Finland
Source: BMC Infect Dis. 2023 Jan 23;23:45. doi: 10.1186/s12879-023-07999-2 (PMC9869503; doi:10.1186/s12879-023-07999-2)
Supplement: Supplementary file 1 — Additional file 1: Table S1. All statistical analyses performed. [file 12879_2023_7999_MOESM1_ESM.docx]

|  | **Correlation to unfavorable outcome (n=60)** | **p-value of univariate analysis** |
| --- | --- | --- |
| **Variable** |  |  |
| Headache | Yes | p=0.0001 |
| Decreased general condition | Yes | p=0.0001 |
| Head CT | Yes | p=0.0001 |
| Hypertension | Yes | p=0.0002 |
| Altered mental status | Yes | p=0.0002 |
| Confusion | Yes | p=0.0011 |
| Operative treatment | Yes | p=0.012 |
| Focal neurological symptoms | Yes | p=0.023 |
| Prediagnostic antibiotic use | Yes | p=0.026 |
| Oral antibiotics on discharge | Yes | p=0.039 |
| Head MRI | Yes | p=0.040 |
| Type 2 diabetes | No | p=0.061 |
| Blood culture positivity | No | p=0.066 |
| Prediagnostic corticosteroid | No | p=0.070 |
| Bacteria cultured from other sources | No | p=0.10 |
| Nosocomial meningitis | No | p=0.11 |
| Pulmonary infections | No | p=0.11 |
| Signs of elevated intracranial pressure with imaging | No | p=0.11 |
| Previous surgery | No | p=0.20 |
| Tobacco use | No | p=0.24 |
| Upper respiratory infection | No | p=0.24 |
| Multi-drug resistance of CSF pathogens | No | p=0.31 |
| Skin infections of the body | No | p=0.33 |
| Acute otitis media | No | p=0.34 |
| Gender | No | p=0.35 |
| Asthma | No | p=0.36 |
| CSF culture positivity | No | p=0.38 |
| Alcohol overuse | No | p=0.39 |
| Acyclovir | No | p=0.44 |
| Seizures prior admission to hospital | No | p=0.58 |
| Dental infections | No | p=0.59 |
| Fever | No | p=0.59 |
| Cancer | No | p=0.61 |
| Skin infections of the head | No | p=0.73 |
| Corticosteroid use | No | p=0.74 |
| First contacted healtcare provider | No | p=0.80 |
| Vomiting | No | p=0.86 |
| Skin color changes | No | p=0.87 |
| Sinusitis | No | p=0.90 |
| Neck stiffness | No | p=0.92 |
| Signs of meningitis with imaging | No | p=1.00 |
|  |  |  |
| **Other statistical analyses** |  |  |
| Pre-diagnostic antibiotic use was statistically associated with negative CSF culture (p=0.05) | |  |
| With positive CSF culture occurred significantly more positive blood culture (p=0.0001) | |  |
| Pre-diagnostic antibiotic use was statistically associated with negative blood culture (p=0.01) | | |
| In age group of 46-65 years positive blood cultures occurred statistically more often than in other age groups (p=0.008) | | |
| CSF-culture positivity was not statistically associated with age groups (p=0.69) | |  |

Table S1. To find out factors affecting to unfavorable outcome (GOS scores 1-4), log binomial model was performed separately for each factor (univariate approach) and reported with relative risk (and it’s 95% confidence intervals) together with p-value. Association with two categorical variables was tested with Fisher’s exact test.
